# Is there an accurate relationship between simple self-reported functional limitations and the assessment of physical capacity in early old age?

**DOI:** 10.1371/journal.pone.0211853

**Published:** 2019-03-08

**Authors:** Valérie Amiard, Jean-Pierre Libert, Alexis Descatha

**Affiliations:** 1 Service de médecine du travail Centre hospitalier Philippe Pinel, Dury, France; 2 UMR-101 PERITOX Unité mixte INERIS, Présidence UPJV Chemin du Thil Amiens, France; 3 Univ of Versailles St-Quentin (UVSQ), UMS 011, UMR-S 1168, Villejuif, France; 4 Inserm, Population-Based Epidemiological Cohorts Unit, UMS011, Villejuif, France; 5 Inserm, Aging and chronic diseases. Epidemiological and public health approaches, U1168, Villejuif, France; 6 Univ Angers, CHU Angers, Univ Rennes, Inserm, EHESP, Irset (Institut de recherche en santé, environnement et travail)—UMR_S1085, Angers, France; 7 AP-HP (Paris Hospital “Assistance Publique Hôpitaux de Paris”), Occupational Health Unit EMS (Samu92), University hospital of West Suburb of Paris, Poincaré site, Garches, France; Berner Fachhochschule, SWITZERLAND

## Abstract

**Study design:**

Observational study.

**Objective:**

To assess the relationship between individual self-reports and measurements of physical condition in early old age.

**Background:**

The use of self-reported questions assessing physical limitations remains questionable in large epidemiological studies. We aimed to test whether there is an accurate relationship between objective measures of physical capabilities and answers given to questions asked of general early old age populations.

**Methods:**

20,335 subjects (45 to 69 years old) performed two gait speed tests at usual and at rapid speeds, and a hand grip strength test. They also completed an interview which included questions about general and specific limitations on their ability to walk one kilometer, climb stairs, and carry 5 kg over a distance of 10 meters. The questions were coded by the patients on a 4-point scale according to the severity of the limitation. Analyses were performed using description of distributions and related tests were carried out.

**Results:**

A fair association was found between individual self-reports and measurements of physical state: limitations on walking one kilometer and climbing stairs were more closely related to rapid than to usual gait speed and to carrying a 5 kg load. For general limitations, the strength of these associations was weaker than the other scores. The association between hand grip strength and the reported score for carrying a mass was better than that for gait speed tests.

**Conclusion:**

Such simple self-assessment questions on physical performance might be useful tools for evaluating functional limitations across a large early old age population in epidemiological research.

## Introduction

In 2011, for the European population, the life expectancy at age 65 was 18 years for men and 21.4 for women. Life expectancy depends on age and gender but provides only limited information on longevity, or quality of life, which is influenced by the individual’s state of health and functional abilities. The physical decline process follows a continuous pattern and ends with disability and dependence [1]. As a result, measures of physical capabilities could be relevant markers for diseases and general health status. In the clinical situation, gait speed is a simple and reliable measure of physical performance, quick to assess, inexpensive, with high test and retest reliability [1,2,3], and informative across multiple clinical and research settings [4,5,6,7]. Gait speed reflects limb strength [6, 8] and mobility and has been shown to be useful for predicting adverse clinical outcomes [2,4,9]. Various studies have also found associations between physical capability, investigated by grip strength [10, 11] and gait speed [6, 12], and all causes of morbidity. Grip strength is thus a predictive technique, and low values are associated with falls disability [13, 14, 15] impaired health [16, 17] and quality of life as well as increased mortality. Interest in hand grip strength and ability to walk is increasing, and the use of both as screening tools to identify people in a population who may benefit from targeted interventions such as strength training or drug treatments [18] is recommended for the measurement of muscle function.

Even though walking speed and hand grip tests can be performed on a given subject, their feasibility for evaluating health damage across a large population remains difficult. Measuring physical performances of patients calls for trained experimenters, special equipment, and calibration of instruments, and is also time- consuming. Moreover, the 4-meter gait speed test, which is the most current tool, cannot always be carried out on patients with severe physical limitations, since it requires effort from older patients, and there is an increased risk of falling or adverse effects related to thoracic and muscular pain. This can disqualify a number of patients [12]. One possible alternative is to reduce the walking distance but the reproducibility of walk tests shorter than 4 meters is not well documented [19]. Another approach uses self- reported assessments of physical limitations. The use of questionnaires is easier, simpler, and inexpensive, but is still criticized. [20] pointed out that self-reported responses may give information of prognostic value, but can be strongly influenced by confounding factors related to environmental conditions, job activity, cultural preferences, or attitude, which impede comparisons across populations and subgroups [21]. Slower gait speed was also less frequent in manager/executive patients than in other occupational categories of workers [22]. Reuben et al. [18] have focused on functional limitations and especially disability, and concluded that it is not possible to identify individuals in an early stage of impairment. Occupational and personal factors can also influence gait speed [19, 23]. Thus each of these quantitative and subjective methods for measuring physical limitations has its advantages and drawbacks. To our knowledge, there is no study which has examined the links between self-reported and quantitative measurements of physical limitations.

The most frequently used questionnaires concern limitations on performing basic daily activities such as bathing or eating, and mobility such as walking or raising one’s arm. The present study aims to investigate whether there is an accurate relationship between objective quantitative measurements and self-reported physical limitations. It uses both general questions on health and routine activities and specific questions referring to various activities such as climbing stairs, walking, and carrying a load in order to test the robustness of the connections between subjective and objective responses. The goal was to determine if there is an accurate relationship between simple self-reported functional limitations and the assessment of physical capacity in early old age for epidemiological research.

## Methods

### Participants

The present study is part of the national CONSTANCES cohort, which was designed as a representative sample for age, gender, and socio-economic status of the French adult population aged 18 to 70. The general design is detailed elsewhere [24,25]. However, due to our partnership with the National Health Insurance Fund administered by the “Caisse Nationale d’Assurance Maladie des Travailleurs Salariés” (CNAMTS), the source population was restricted to salaried workers, professionally active or retired (more than 85% of the French population, i.e. approximately 50 million people), excluding agricultural and self-employed workers who are affiliated with other health insurance funds.

In France, all those with health insurance from CNAMTS, as well as their dependents, receive free health examinations that include extensive checkups conducted at Health Screening Centers (HSCs). Overall the 110 HSCs perform approximately 500,000 health examinations annually. Thanks to our partnership with CNAMTS, we include the cohort participants in 22 selected HSCs located in the principal regions of France. Randomly selected eligible subjects (see below) were invited to come to their HSC. The volunteers completed questionnaires at home before attending their HSC where they signed an informed consent and underwent a health examination.

Data concerning social and demographic characteristics, health-related behaviors and health status are collected regularly by trained investigators using different sources (questionnaires, medical examinations, national health and social databases).

The main exclusion criteria were: refusal to participate, handicap, visible upper limb deformity, arthritis, amputation, significant pain, a recent accident, or hand surgery in the previous six months. The analysis was restricted to those able to perform all the required experimental tasks. The detailed English version of the inclusion and follow-up data catalogue can be downloaded from the CONSTANCES website [24,25].

All confidentiality, safety and security procedures were approved by the French legal authorities. In accord with French regulations, the CONSTANCES cohort project has obtained the authorization of the National Data Protection Authority detailed in previous studies [26,27,28].

### Self-assessment questionnaire on limitations

This questionnaire was validated for this population [24,25,26]^.^ It included 4 different levels of limitations measured on a 4-point scale as follows:

A general question: “Over at least the past 6 months, have you been limited, i.e. do you experience difficulties due to a health-related problem, in performing routine activities (at home, at work, leisure activities, etc.) in comparison with other people of your age?” The responses were encoded as: Yes; significantly limited; Yes, limited; Yes, but slightly limited; or No.

A question on climbing stairs: “Are you able to walk up or down a flight of stairs alone?” encoded as: Yes, without difficulty; Yes, but with some difficulty; Yes, but with significant difficulty; No.

A question on walking: “Can you walk one kilometer alone, without stopping and without being seriously inconvenienced?” encoded as: Yes without difficulty; Yes, but with some difficulty; Yes, but with significant difficulty; No.

A question on physical work: “Are you able to carry a 5 kg weight over a distance of 10 meters (e.g.: a shopping bag, a school bag)?” encoded as: Yes, without difficulty; Yes, but with some difficulty; Yes, but with significant difficulty; No.

### Quantitative measurements of physical limitations

Two timed gait tests were successively performed at maximum and usual normal speeds over a short distance of 3 meters. The participant had to walk from a standing position between two parallel, three-meter-long straight lines clearly marked on the ground, and then stop just after the end of the path. Additional zones of one meter before and after the start and stop lines allowed them to accelerate and decelerate. The instructions given were: “we will perform two walking-time tests. The first will be done at your usual walking speed, the second as quickly as possible”.

To ensure that all the participants had correctly understood the procedure, the investigator explained each part of the test and demonstrated it as often as necessary. A training trial was made before measurement. For safety reasons, the investigator walked beside but slightly behind the patient (without touching him/her) in case the latter might trip or lose his/her postural balance. The stopwatch was started when the first foot had fully crossed the start line and stopped when one foot had completely crossed the finish line and touched ground on the far side of the finish line. The whole foot had to cross the finish line. Again, if the participant dragged his/her feet or stepped on the finish line, the stopwatch was stopped once the foot had completely crossed the line. Gait speed was expressed in seconds.

The Hand grip test was performed with a JAMAR portable Hand Dynamometer (Lafayette Instrument Company, USA, precision of 0.1 Kg) currently accepted as a gold standard for handgrip strength [29]. An experimenter performed 3 successive trials for each hand using the standard procedure describe below. The average values of these trials were compared to the normative data defined by [30] and scores within 2 standard deviations were considered normal. The patient was invited to remove any rings and the following information was given: “We are going to test your grip strength. I am going to ask you to squeeze this handle as hard as you can for two seconds and then relax for one minute”. The patients were asked to perform the task three times for each hand. If measurements with one hand were problematic, only the values recorded for the other hand were taken into account, and the results were recorded accordingly. The dynamometer’s adjustable handle was set to the desired spacing, so that it fitted comfortably in the patient’s hand, the handle resting on the second phalanx of the index finger and the adjacent fingers. After the spacing had been locked, the sitting patient held the dynamometer with the shoulder adducted and neutrally rotated, the elbow flexed at 90° and the (unsupported) forearm in a neutral position. The dorsiflexion of the wrist was between 0 and 30°. The peak-hold needle had to be rotated to the zero position. The patient was then instructed to "squeeze the handle as hard you can". The peak-hold reading (in kilograms^-1^) was recorded, and the needle was reset to zero. Any protocol deviations were noted in the patient’s case report form.

### Statistical analysis

All variables were recorded to have lower value for lower limitation and higher value for higher limitation. Since higher values for the hand-grip results originally corresponded to lower limitation, these results were inverted (i.e. computing the inverse of the hand grip result) to have a similar trend between the other limitation evaluations. Then, all limitations variables are coded from the lowest to the highest limitations.

Distribution of the data and associations between each of the four categories of self-reported limitations and the gait speed and hand-grip tests were described using mean with standard deviation, median and maximal and minimal values of each variable.

General linear models were created for analyzing variance and the association between self-reported limitations and the gait speed and hand-grip tests, both without and with adjustments for gender, body mass, size and age.[21] P and F-values were given.

Since multiple comparisons can increase the problems of intercorrelation between tests and inflate the probability of type 1 errors, the accepted level of significance was reduced to 0.01. Statistical analyses were performed using Statistic Analysis Software SAS V9.4 package (SAS Institute, Inc. Cary, NC, USA).

## Results

Demographic details of the recruited population have been previously provided elsewhere [24,25] In the present study the sample included 20,335 patients who carried out all the tests. The sample included 10,644 women (52.4%) and 9,681 men (47.6%). At the time of the survey, the age percentages were 16.4% for 45–50, 19.6% for 50–55, 20.3% for 55–60, 20.8% for 60–65, and 22.9% for 65–69. The self-reported scores are reported in Tables 1–3;14.5% of the subjects reported general limitations, 7.5% limitations for climbing stairs, 6.6% for walking one kilometer alone, and 9.2% for carrying a mass of 5 kg over 10 meters.

The times recorded during the normal (Table 1) and rapid (Table 2) walk tests were significantly higher (p≤ 0.001) for the patients who reported limitations *versus* no limitations. Depending of the type of self-reported limitations, normal gait speeds varied from 2.50 seconds when patients did not report limitations to 2.69–2.90 second in case of limitations (t always ≥ 19.87). This was also observed for rapid gait speed (1.65 vs 1.82–2.00 seconds; t always ≥ 24.49). The significant F-values indicate that the time gait speed was strongly associated with the 4 levels of the self-reported scores of limitation taking into consideration the two gait speed tests. The larger the self-reported scores of limitations, the longer it took to perform the test and thus the slower the time gait speeds.

**Table 1:** Normal gait speed measures (in second) and self-reported scores referring to general limitation, climbing stairs, walking one kilometer and carrying 5 kg over a distance of 10 meters.

		n	Mean	SD	MED	MIN	MAX	Test 1	Test 2
**Global limitation**	No	12.916	2.48	0.44	2.43	0.94	7.34	F = 555.23***	F = 303.15***
	slightly	4.460	2.57	0.48	2.53	1.00	6.00		
	limited	2.241	2.66	0.54	2.59	0.93	6.23		
	significantly	718	2.79	0.73	2.67	0.94	10.00		
	No	17.376	2.50	0.45	2.45	0.94	7.34	t = -19.87***	
	Yes	2.959	2.69	0.60	2.61	0.93	10.00		
**Climbing stairs**								F = 630.90***	F = 349.32***
	No	18.811	2.51	0.45	2.45	0.93	7.34		
	slightly	1.346	2.82	0.64	2.74	0.94	10.00		
	limited	107	3.29	0.94	3.10	1.83	7.70		
	significantly	71	2.59	0.47	2.50	1.86	4.38		
	No	18.811	2.51	0.45	2.45	0.93	7.34	t = -26.88***	
	Yes	1.524	2.85	0.67	2.75	0.94	10.00		
**Walking** **1 km**								F = 934.09***	F = 561.74***
	No	19.001	2.51	0.46	2.45	0.93	7.34		
	slightly	1.071	2.83	0.58	2.76	1.23	5.64		
	Limited	143	3.09	0.70	2.97	1.91	6.30		
	significantly	120	3.23	1.10	3.02	1.85	10.00		
	No	19.001	2.51	0.46	2.45	0.93	7.34	t = -29.01***	
	Yes	1.334	2.90	0.67	2.80	1.23	10.00		
**Carrying 5 Kg**								F = 708.03***	F = 362.03***
	No	18.461	2.51	0.45	2.45	0.93	7.34		
	slightly	1.512	2.75	0.58	2.68	1.26	6.23		
	limited	217	2.98	0.70	2.88	1.65	6.66		
	significantly	145	3.01	0.97	2.84	1.86	10.00		
	No	18.461	2.51	0.45	2.45	0.93	7.34	t = -25.50***	
	Yes	1.874	2.80	0.64	2.71	1.26	10.00		

Mean with standard deviation (SD), Median (MED), Minimum (MIN), Maximum (MAX) values and the number of patients (n) are indicated. F-values reported in “test 1 and 2” are respectively found without and with adjustment for gender, body mass, size and age.

Statistical comparisons between patients who do or do not report, limitations (No *vs* Yes) are also indicated (t-values).

*** p<0.0001.

**Table 2:** Rapid gait speed measures (in second) and self-reported scores referring to general limitation, climbing stairs, walking one kilometer and carrying 5 kg over a distance of 10 meters.

		n	Mean	SD	MED	MIN	MAX	Test 1	Test2
**Global limitation**	No	12.916	1.64	0.30	1.64	0.48	4.02	F = 817.51***	F = 475.73***
	slightly	4.460	1.71	0.33	1.70	0.29	4.00		
	limited	2.241	1.79	0.39	1.76	0.64	4.08		
	significantly	718	1.92	0.60	1.85	0.29	8.90		
	No	17.376	1.65	0.31	1.65	0.29	4.02	t = -24.49***	
	Yes	2.959	1.82	0.45	1.77	0.29	8.90		
**Climbing stairs**								F = 1077.45***	F = 639.25***
	No	18.811	1.65	0.31	1.65	0.29	4.02		
	slightly	1.346	1.96	0.50	1.91	0.69	8.90		
	limited	107	2.32	0.73	2.16	1.00	6.64		
	significantly	71	1.71	0.34	1.65	1.00	2.87		
	No	18.811	1.65	0.31	1.65	0.29	4.02	t = -35.87***	
	Yes	1.524	1.97	0.52	1.91	0.69	8.90		
**Walking** **1 km**								F = 1535.93***	F = 974.32***
	No	19.001	1.66	0.31	1.65	0.29	4.02		
	slightly	1.071	1.95	0.43	1.93	0.56	4.40		
	Limited	143	2.20	0.57	2.10	1.00	4.31		
	significantly	120	2.28	0.91	2.12	1.00	8.90		
	No	19.001	1.66	0.31	1.65	0.29	4.02	t = -37.34***	
	Yes	1.334	2.00	0.52	1.95	0.56	8.90		
**Carrying 5 Kg**								F = 1329.62***	F = 634.49***
	No	18.461	1.65	0.31	1.65	0.29	4.02		
	slightly	1.512	1.89	0.41	1.87	0.56	4.29		
	limited	217	2.11	0.52	2.02	1.00	4.40		
	significantly	145	2.12	0.86	1.98	1.00	8.90		
	No	18.461	1.65	0.31	1.65	0.29	4.02	t = -35.34***	
	Yes	1.874	1.94	0.48	1.89	0.56	8.90		

Mean with standard deviation (SD), Median (MED), Minimum (MIN), Maximum (MAX) values and the number of patients (n) is indicated. F-values reported in “test 1 and 2” are respectively found without and with adjustment for gender, body mass, size and age.

Statistical comparisons between patients who report or not limitations (No *vs* Yes) are also indicated (t-values).

*** p<0.0001.

Similarly, measured muscle strength with the hand grip test (Table 3) was weaker for patients who reported general limitation versus patients without limitations and for hand grip measures (0.034 vs 0.038–0.046 seconds; t always ≥ 11.72) and decreased with the severity of this estimation (significant F-values).

**Table 3:** Inversed hand grip measures (in kilogram^-1^) and self-reported scores referring to general limitation, climbing stairs, walking one kilometer and carrying 5 kg over a distance of 10 meters.

		n	Mean	SD	MED	MIN	MAX	Test1	Test2
**Global limitation**								F = 213.32***	F = 217.33***
	No	12.916	0.034	0,013	0,032	0,012	0.375		
	slightly	4.460	0.036	0.015	0.034	0.014	0.214		
	limited	2.241	0.037	0.017	0.035	0.015	0.30		
	significantly	718	0.040	0.023	0.035	0.014	0.333		
	No	17.376	0.034	0.014	0.032	0.013	0.375	t = 11.72***	
	Yes	2.959	0.038	0.019	0.035	0.014	0.333		
**Climbing stairs**								F = 158.11***	F = 124.52***
	No	18.811	0.035	0.014	0.032	0.012	0.375		
	slightly	1.346	0.040	0.021	0.037	0.015	0.333		
	limited	107	0.046	0.025	0.041	0.018	0.214		
	significantly	71	0.032	0.011	0.028	0.015	0.071		
	No	18.811	0.035	0.014	0.032	0.013	0.375	t = 14.72***	
	Yes	1.524	0.040	0.021	0.037	0.015	0.333		
**Walking** **1 km**								F = 181.85***	F = 149.09***
	No	19.001	0.035	0.014	0.032	0.012	0.375		
	slightly	1.071	0.041	0.020	0.037	0.015	0.333		
	Limited	143	0.042	0.021	0.036	0.018	0.136		
	significantly	120	0.040	0.018	0.036	0.015	0.130		
	No	19.001	0.035	0.014	0.032	0.013	0.375	t = 15.12***	
	Yes	1.334	0.040	0.021	0.037	0.015	0.333		
**Carrying** **5 Kg**								F = 1089.04***	F = 436.37***
	No	18.461	0.034	0.014	0.031	0.013	0.375		
	slightly	1.512	0.045	0.018	0.041	0.015	0.300		
	limited	217	0.050	0.023	0.044	0.018	0.214		
	significantly	145	0.051	0.032	0.044	0.018	0.333		
	No	18.461	0.034	0.014	0.031	0.013	0.375	t = 33.59***	
	Yes	1.874	0.046	0.021	0.042	0.015	0.333		

Mean with standard deviation (SD), Median (MED), min (MIN), Max (MAX) values and the number of patients (n) are indicated. F-values reported in “test 1 and 2” are respectively found without and with adjustment for gender, body mass, size and age.

Statistical comparisons between patients who report or not limitations (No *vs* Yes) are also indicated (t-values).

*** p<0.0001.

## Discussion

The present study shows that physical capability or ability, a term used to describe a person’s ability to perform the physical tasks of everyday life, can be assessed by self-reported estimations compared with quantitative measurements obtained from hand grip and gait speed tests on a 3 m-course. Higher self-limitation reports were significantly associated with poorer physical performance. For self-reports concerning general limitations, the association is weaker. Compared to specific performances such as walking, climbing stairs, or carrying mass, this summary measure, which combines various performance assessments occurring in the last 6 months, is quite general and broader for the patients, covering not just physical limitations. This highlights the relevance of the questions which are asked of the patient.

Before and after adjustments for the potential confounding variables related to gender, age, height, and body mass, self-reported general limitations for walking one kilometer and climbing stairs are associated with gait speed, whereas hand grip is more predictive of physical work associated with carrying heavy loads.

### Gait speed test

Gait speed was measured using a protocol involving a walking distance of 3 meters. This choice was motivated by a technical issue, since 4-meter or higher zones were not available in all places for the examination. Comparison between existing studies in the literature is difficult, since differences in the methods used [distance, acceleration allowed, or stop and go, stopwatch and photocell] are large and not standardized [31, 32]. The reliability and validity of the measures have most often been assessed over a 4-meter course or during 6 and 12 minute walking tests [33] [usually for measuring responses to medical interventions in patients with moderate to severe heart or lung disease]. In our study, the test was designed to cover a short distance, in order to rule out adverse effects related to body imbalance, which can hamper physical performance. This can be relevant for patients who cannot walk long distances, due to physical limitations and/or impaired joints underlying work disabilities. The present data confirm the results of [12] who pointed out that such short tests can be a good predictor of loss of physical function, relevant to the muscle performance of lower body extremities.

### Hand grip test

The hand grip test is a useful functional measure of the integrity of the upper extremity and is the simplest method for assessing upper limb muscle and joint functions in clinical practice [18, 34]. In our study, we used a Jamar hand dynamometer, which is currently accepted as the gold standard [29]. The protocol was standardized in order to improve the precision of the measurements [thereby increasing statistical power to detect associations between grip strength and self-reported limitations]. The item, “are you able to carry a 5 kg weight over a distance of 10 m?” was fairly well associated with hand grip strength. For the physiological processes involved when carrying heavy loads and in the hand grip test, the underlying operational processes are close since similar upper limb muscles and joints are involved. This highlights the relevance of using such tests to assess the functional capabilities of workers who carry heavy loads, a finding which adds a new dimension in the choice of tests to study the impairment of a specific physical function.

### Limitation of the study

With respect to study limitations, it must be considered that the study was performed across a large, geographically diverse population, including volunteers of early old age. The self-reported scores depend not only on each individual history but also on the interpretation both of the different items in the questionnaire and of the rating scale. Other variables would probably be worth considering in further studies, such as the level of sport practice or physical activity at home or at work, since physical fitness influences performance. Melzer et al. [20] have also reported that the subjective components of a questionnaire may give information of prognostic value, but can be strongly influenced by confounding factors related to environmental conditions, job activity, cultural preferences, or attitudes that impede comparisons across populations and subgroups [21]. Conceivably some of the recruited patients were people with diseases or co-morbidities which were not considered severe enough to warrant exclusion from the study but which could affect their physical performance. Another concern relating to possible experimenter influence on patient performances cannot be excluded [35]. Even though the evaluations were undertaken in a standardized way by trained investigators, it cannot be ruled out that not all clinical sites involved uniformly collected self-reported disability information or administered performance-based testing at the same time periods. Thus, seasonal and/or time of day influences on biological rhythms should be considered, since the measurements were done at different periods of the year and times of day. A technological limitation is also conceivable, since it has been shown that gait speed measurements with a stopwatch can be influenced by intra- and inter- experimenter variability [6, 7].

### Conclusion

Despite these various limitations, the present study indicates that a self-reported such questionnaire can be recommended as a proxy for physical limitations across a large population for epidemiological research. The present study shows that self-reported scores on general limitation, ability to walk one kilometer, and climb stairs are closely related to gait speed, especially rapid gait speed measured over a short distance of 3 meters. It should be noted that the discrimination is better with specific questions referring to physical performances such as climbing stairs, walking, and carrying a load, than a general question on health and routine activity problems based on facts occurring during the last 6 months. However, further studies are needed to confirm whether the associations between self-reported and objective measure found in the study are applicable when using other questionnaires.

## Key points

### Finding

In patients of early old age, self-reported limitations on physical functions related to general limitation, to an ability to walk one kilometer, to climb stairs and to carry 5 kg over a distance of 10 meters are more closely related to rapid than to normal gait speed on a 3 meter course.

Hand grip strength was a better indicator of reported performance limitations for carrying a heavy load than gait speeds.

### Implications

Simple self-assessment questionnaires on physical performance are useful tools which can be recommended for research evaluating functional limitations across large populations.

### Caution

The weakness of the association between self-reports referring to a general limitation and quantitative measurements of physical performance in patients at early old age may be due to the generality of this question, which requires that the subject recall all events occurring over the last 6 months, and is not intended to replace objective measures conducted in clinical settings. The results are only applicable to the questionnaires studied here.
